# An Artificial Neural Network Model for Pediatric Mortality Prediction in Two Tertiary Pediatric Intensive Care Units in South Africa. A Development Study

**DOI:** 10.3389/fped.2022.797080

**Published:** 2022-02-25

**Authors:** Michael A. Pienaar, Joseph B. Sempa, Nicolaas Luwes, Lincoln J. Solomon

**Affiliations:** ^1^Paediatric Critical Care Unit, Department of Paediatrics and Child Health, University of the Free State, Bloemfontein, South Africa; ^2^Department of Biostatistics, Faculty of Health Sciences, University of the Free State, Bloemfontein, South Africa; ^3^Department of Electrical, Electronic and Computer Engineering, Faculty of Engineering, Built Environment and Information Technology, Central University of Technology, Bloemfontein, South Africa

**Keywords:** critical care, children, severity of illness, machine learning, artificial neural network

## Abstract

**Objectives:**

The performance of mortality prediction models remain a challenge in lower- and middle-income countries. We developed an artificial neural network (ANN) model for the prediction of mortality in two tertiary pediatric intensive care units (PICUs) in South Africa using free to download and use software and commercially available computers. These models were compared to a logistic regression model and a recalibrated version of the Pediatric Index of Mortality 3.

**Design:**

This study used data from a retrospective cohort study to develop an artificial neural model and logistic regression model for mortality prediction. The outcome evaluated was death in PICU.

**Setting:**

Two tertiary PICUs in South Africa.

**Patients:**

2,089 patients up to the age of 13 completed years were included in the study.

**Interventions:**

None.

**Measurements and Main Results:**

The AUROC was higher for the ANN (0.89) than for the logistic regression model (LR) (0.87) and the recalibrated PIM3 model (0.86). The precision recall curve however favors the ANN over logistic regression and recalibrated PIM3 (AUPRC = 0.6 vs. 0.53 and 0.58, respectively. The slope of the calibration curve was 1.12 for the ANN model (intercept 0.01), 1.09 for the logistic regression model (intercept 0.05) and 1.02 (intercept 0.01) for the recalibrated version of PIM3. The calibration curve was however closer to the diagonal for the ANN model.

**Conclusions:**

Artificial neural network models are a feasible method for mortality prediction in lower- and middle-income countries but significant challenges exist. There is a need to conduct research directed toward the acquisition of large, complex data sets, the integration of documented clinical care into clinical research and the promotion of the development of electronic health record systems in lower and middle income settings.

## Introduction

Prediction of mortality in the pediatric intensive care unit (PICU) has applications in clinical care. The most prevalent application for such predictive models in the PICU setting is standardized evaluation of PICU services by evaluating observed mortality against predicted mortality (1–7). Reliable prognostic estimates may also allow providers to inform families of the likely outcome of patients admitted to intensive care units although limitations in the performance of current models at an individual level hamper their use in patient care (8). Predictive models may also be useful to evaluate usage of scarce resources by identifying futility (9). This goal is highly relevant in resource limited settings, where ensuring equitable and justifiable access to limited resources in a key concern (10, 11).

Currently the Pediatric Index of Mortality 3(PIM3) is applied in our setting to monitor unit performance (2). The application of several existing mortality prediction scores has been investigated in lower income countries (6, 12–15). Model performance has been variable in these settings with calibration and discrimination having been cited as challenges (12, 13, 16, 17). Beyond this, the clinical applications of such models in these settings are constrained by the large numbers of variables or the necessity for laboratory values within these models (15). It has been proposed that miscalibration indicated by significant Hosmer Lemeshow(HL) *p*-values are likely due to better or worse standards of care in the evaluated PICU as compared to the development study group (18). In their recent evaluation of PIM3 in South Africa, Solomon et al. found PIM3 to be poorly calibrated in a multicenter prospective study in South Africa. They suggested that significant HL *p*-values in South Africa may be due to case-mix differences between the studied population and the derivation population (13). This supports the need investigate and develop such models in these settings. In this study we investigate ANNs as a novel method for this application, but also apply the standard method of logistic regression, both to the development of a new model and a recalibration of an existing standard model (PIM3).

Machine learning techniques for a range of biomedical applications have become prevalent in the medical literature. These techniques emphasize learning from available data and exist on a spectrum defined by the degree of human involvement and the reciprocal autonomy of computer systems in the operations of these algorithms. This spectrum includes conventional statistical approaches such as logistic regression models with high levels of human input on one end, with increasing levels of computer autonomy in classical machine learning and high levels of computer autonomy in deep learning models such as convolutional neural networks (19). Advanced machine learning algorithms offer an ability to engage with complexity and non-linearity that make them an appealing tool for a wide range of new applications such as image recognition with convolutional neural networks (20) and time series analysis with recurrent neural networks (21) and existing applications such as prediction of end-points such as mortality (22). Recently, several studies have reported the use of machine learning approaches with encouraging results (21, 23–25). To date, the full extent to which machine learning approaches can be applied in pediatric research has not been fully explored, but, as Londsdale et al. point out, the opportunities for improving patient care are substantial (26).

Artificial neural networks are computational structures designed to emulate the organizational functioning of biological neurons (27). Connected input, hidden and output layers make up the structure of artificial neural networks. Numerical values, called weights, determine the strength of connection between neurons in each layer. Outputs of neurons are determined by a mathematical function, the activation function that takes in the inputs to input neurons, weights of connections and threshold terms of each neuron (28). Artificial neural networks used in supervised classification tasks such as described in this study are referred to as perceptrons. Weights are adjusted through stochastic gradient descent (25).

An accurate and complete assessment of the predictive performance of developed models is a crucial step in their development. Performance is most often reported in terms of discrimination (the receiver operating characteristic curve -ROC and the area under the receiver operating characteristic curve – AUROC). These metrics provide a model-wide, visually interpretable view of model performance. While ROC and AUROC are the most reported metrics of model performance, their use in isolation may lead to optimistic reports of model performance in imbalanced data sets such as can be expected in most mortality prediction models. For this reason, the precision-recall plot (PRC) and area under the curve for the PRC (AUPRC) may be more informative metrics for the evaluation of such models. The PRC relates precision (also called positive predictive value [True Positives/(True Positives + False Positives)] and recall (also called sensitivity; True Positives/[True Positives + False Negatives)]. And provides a model-wide evaluation of performance. The AUPRC, similarly allows comparisons between models. As opposed to the AUROC where the baseline value for random classifiers is 0.5. The value for random classifiers in the case of the AUPRC is not fixed, but rather corresponds to the proportion of positive class [Positive/(Positive + Negative)]. The AUPRC of a perfect classifier is 1.0. (29). Calibration is a frequently neglected performance metric (30–33). This metric refers to the concordance between predicted probabilities of models and real probabilities of the investigational event (34). The utility of mortality prediction models for quality auditing, counselling of family, risk stratification in research or for evaluation of rationing of services is related to a reliable fit between posterior probabilities and the real likelihood of the outcome. Van Calster and Vickers demonstrated with simulation data, that miscalibrated models degraded Net Benefit of models and even caused harm where models underestimated risk at a threshold below the event rate or overestimated risk at a threshold above event rate (32). Van Calster et al. suggest that the calibration of models can be characterized in the mean (average probabilities vs. event rate), weak (calibration intercept and calibration slope), moderate (the closeness of a flexible calibration curve to the diagonal) and strong calibration (the utopic, perfect or near perfect calibration of predictions to event rates for all categories of prediction) (34). This study followed the Transparant Reporting of a Multivariable Prediction Model for Individual Prognosis or Diagnosis (TRIPOD) guidelines (35).

In this exploratory study, we identified prognostic factors for mortality in the PICU and used these features to develop ANN and LR models for PICU mortality prediction. The features collected were those previously used in PIM3 and features relevant to the data set were determined by a wrapper algorithm in the case of the ANN and by multivariate logistic regression for the LR model. The LR model, a recalibrated version of PIM3 the calculated PIM3 score from the original model were compared to the performance of the ANN model.

## Methods and Materials

### Ethics Approval

Internal review board approval of this study was obtained from Health Sciences Research Ethics Committee of the University of the Free State (UFS – HSD2021/0091/2906) and the Free State Department of Health (FS202102 019).

### Objectives

The objective of this study was to explore the use of ANNs as a machine learning method for mortality risk prediction in PICU in a lower-middle income (LMIC) setting. This was achieved through the following subobjectives: (1) An ANN model was developed, trained, and tested without the use of advanced computing infrastructure. (2) The performance of this model was evaluated and compared to the performance of an LR model (as a common, well-recognized method used for this application) developed on the same data set as well as a version of PIM 3 recalibrated on this data set.

### Study Setting

Patients from two tertiary hospitals were included in this retrospective cohort study. Patients under the age of 13 completed years were admitted to the PICU of Pelonomi Tertiary Hospital and Universitas Tertiary Hospital from January 2017 to January 2021. Each of these PICUs have 5 beds, providing life supporting care including advanced monitoring, high-flow nasal oxygen, non-invasive ventilation, conventional ventilation, high frequency oscillatory ventilation, vasoactive medication, renal replacement therapy and neuroprotective care including intracranial pressure monitoring. Each of these PICUs admit ~250 patients per annum. Clinical staffing includes a paediatric intensivist and paediatric critical care trainee together with paediatric specialty trainees and paediatric hospitalists after hours. These centralized PICUs provide paediatric critical care services to the state sector primarily in the Free State Province of South Africa but also advanced services on a referral basis to patients from the Northern Cape Province and Lesotho. A wide spectrum of patients are provided care in our setting. Pelonomi Hospital provides predominantly care for acute illness and trauma, including burns, but also provides perioperative care for paediatric surgery, neurosurgery and spinal surgery. Universitas Hospital provides predominantly peri-operative care (paediatric surgery, neurosurgery, orthopaedic surgery, ophthalmology, otorhinolaryngology and urology) and paediatric subspecialty referrals (including paediatric oncology and cardiology). Referrals include patients from within the two hospitals, either from Emergency Department, paediatric wards or operating theatre as well as referrals from remote facilities. Patients include patients from urban, peri-urban and rural populations and are generally uninsured. Emergency transport services are limited in our setting, with one operating ambulance capable of intensive care transportation, one helicopter ambulance across the province. Emergency services are also limited, with no dedicated paediatric resuscitation service or dedicated Emergency Medicine specialty department.

### Approach

The approach employed in this study is one of a wide range of approaches to the highly complex task of mortality prediction. In our setting, PIM3 has been employed since 2017 as part of quality control monitoring and benchmarking. PIM3 offers the advantage of only requiring a single data collection on admission to the PICU. It, however, does not predict time to death or make use of changing time series data, which are limitations. Approaches capable of making dynamic continuous predictions of PICU mortality using convolutional or recurrent neural networks have been described (23, 24). In our setting, however, we currently do not have a means of collecting continuous time series data from patients as these recordings are still recorded by hand at the bedside. As a result, the largest dataset is that is available to us is from our use of PIM3. This approach does however allow investigation of a novel method with the same advantage of PIM3 and lay a further foundation for developing strategies to make machine learning viable in critical care environments in LMICs.

### Study Population and Sampling

The sample included all 2,089 records in the data set. Necessary sample size was determined both in terms of the total required sample size and the required events per variable recommended for logistic regression. Applying the rule of thumb of a minimal sample size of 500 and *n* = 100+ 50*i* where *i* is the final number of independent variables in the model (36), a sample size of 2,089 records could accommodate a model of up to 39 variables from the perspective of total sample size. In terms of events per variable (in this instance events refer to the number of deaths), Peduzzi et al. (37) suggest that above 10 events per variable were not associated with problems in logistic regression analysis while Bujang et al. (36) suggest the requirement of 50 events per variable. With 226 non-survivors after removal of duplicate records and records with missing data, between 4 and 22 variables could be included in a viable model.

### Data Collection

At present there are limited capabilities for the collection of electronic health data in our setting, with only laboratory results, radiology reports, summary clinical reports and administrative and billing information available on the electronic health records of the hospitals. No continuous recording of clinical care is available on the system. An available data set existed in the form of the data that had been collected for use in the calculation of PIM3 scores as part of bench-marking and quality controlled. This data was collected during the first hour of admission to the PICU by clinicians working in the PICU, directly into a password protected spreadsheet during clinical care. The variables available in this data set are presented in Table 1. Low-risk, high-risk and very-high-risk diagnostic categories are those identified and validated in the original PIM3 model (2). No further data was collected. Data was anonymised by deletion of all identifying data and directly imported to a secure REDCap® database (38).

**Table 1:** Variables collected.

**Variable**		**Data type**
Age		Continuous
Systolic blood pressure (mmHg)		Continuous
Pupil reactions to bright light (>3mm and fixed, unresponsive)		Binary
Absolute base excess in arterial blood		Continuous
Partial pressure of oxygen in arterial blood (PaO_2_) (mmHg)		Continuous
Fraction of inspired oxygen(FiO_2_) (decimal)		Continuous
([FiO_2_ x 100]/PaO_2_)		Continuous
Recovery from non-cardiac procedure		Binary
Recovery from non-bypass cardiac procedure		Binary
Recovery from bypass cardiac procedure		Binary
Low risk diagnosis^*^: Asthma Bronchiolitis Croup	Obstructive sleep apnoea Diabetic ketoacidosis Seizure disorder	Binary
High risk diagnosis^*^ Spontaneous cerebral haemorrhage Cardiomyopathy or myocarditis Hypoplastic left heart syndrome	Neurodegenerative disorder Necrotising enterocolitis	Binary
Very high risk diagnosis^*^ Cardiac arrest preceding ICU admission Severe combined immunodeficiency Leukaemia or lymphoma after first induction	Bone marrow transplant recipient Liver failure is the main reason for ICU admission	Binary

* *Diagnostic risk categories from PIM3 (2)*.

For descriptive analysis, a Student's t-test was used to compare the means between survivors and non-survivors for numerical variables, and Chi-Square test for categorical variables.

### Logistic Regression Model and Recalibration of PIM3

Multivariate Logistic regression was used to determine factors associated mortality in the pediatric intensive care units. Variables which are known confounders and those that were statistically significant relationship in the bivariate analysis were included. Results were summarized into adjusted Odds Ratio (Adj. OR), 95% Confidence and Interval (95% CI). A statistically significant relationship was one with a *P* < 0.05. For model calibration purposes, we split the dataset into two, i.e., 70% for training and 30% for and testing, and, in each case, estimated the AUROC and calculated the Hosmer-Lemshow test for the final multivariate logistic regression model. Statistical analysis was performed using R, version 4.0.2 (39).

### Artificial Neural Network Model

A commercially available desktop computer was used in the development of the ANN model. The artificial neural network model was developed using Python 3 (40) within the Jupyter Notebooks environment (41). NumPy (42), Pandas (43), Scikit-learn (44), Tensorflow (45), Keras and SciPy libraries were used in the development of the artificial neural network model.

The data was further preprocessed for the development of the artificial neural network model. We removed duplicated records and those with impossible or missing values as there were only 70 such records.

Systolic blood pressure was categorised using the age-related threshold values according to the Pediatric Logistic Organ Dysfunction scoring system in the 10- and 20-point variable categories (46). The data was randomly split into a training set and test set in an 70/30 split. A wrapper algorithm was employed to select features from the training data, associated with the target with a *p* < 0.05. The following features were included: pupils unresponsive to light, elective admission, mechanical ventilation in the first hour of admission, categorised blood pressure below the 10-point category, absolute value of base excess (ABE), fraction of inspired oxygen (FiO_2_), the ratio of partial pressure of oxygen in arterial blood to fraction of inspired oxygen (PF), very-high-risk diagnosis (from PIM3 model), high-risk diagnosis and low-risk diagnosis. Features were scaled using MinMax scaling.

### ANN Development, Training, and Testing

Due to the small size of the data, a third validation set for model tuning was thought unfeasible and thus, K-fold cross validation with 10 folds was used in the training phase. We optimised model hyperparameters using a randomized grid search. The neural network model developed was a simple feed-forward multi-layered perceptron with two densely connected hidden layers of 15 neurons with a dropout layer with a dropout rate of 0.1 after each hidden layer. We made use of a rectified linear unit (ReLU) activation function in the hidden layers and a sigmoid activation function in the output layer. We used a batch size of 80 and 100 epochs. Cross validation was then undertaken prior to training the model on the training data.

### Model Performance Evaluation

**Figure 1:**
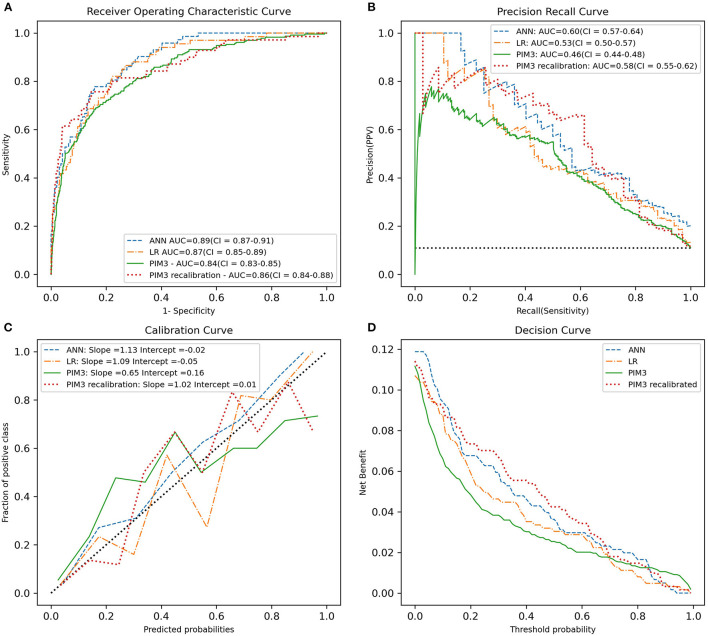
Predictive performance of the ANN, LR, recalibrated PIM3 and original PIM3 models. **(A)** The ROC curve. **(B)** The precision-recall curve. **(C)** The calibration cure. **(D)** The decision curve. The area under the curve in A and B and the slope of the calibration curve in C are shown in the legend. PIM3 performance metrics were calculated across the whole data set, while the ANN and LR performance metrics were calculated on the test set.

For the results on the test set we plotted the ROC curve, PRC curve, calibration curve and decision curve and calculated the AUROC, AUPRC, mean squared error (MSE), slope and intercept of the calibration curve and the HL statistic (see Figure 1). This was done for the ANN models and LR model. PIM3 was also assessed over the whole data set.

## Results

A descriptive analysis of the data collected is presented in Table 2. There was significant imbalance between the majority class (survivors) and the minority class (non-survivors). There were 1,793 survivors and 226 (10.81%) non-survivors in the data set. The mean age of survivors was 43.16 months (standard deviation 49.98) and 36.74 (standard deviation 46.67) in non-survivors (*p*-value 0.063). There were 159 mortalities in the training data and 67 in the test data. There were 334 planned admissions and 1,755 unplanned admissions in the data set. Surgical procedures preceded admission to the PICU in 344 cases. This included 10 bypass cardiac surgeries, 10 non-bypass cardiac surgeries and 324 non-cardiac procedures.

**Table 2:** Descriptive analysis.

**Variable**	**Total**	**Alive**	**Dead**	***P*-value**
Age of the respondents; mean (SD)	2,086	43.16 (49.98)	36.74 (46.67)	0.0627
Absolute value of base excess (mmol/l): mean (SD)	2,089	7.07 (6.71)	11.53 (8.68)	<0.0001
SBP (mmHg): *Mean (SD)*	2,062	101.21 (23.85)	88.14 (27.94)	<0.0001
**Pupils fixed to light:** ***n (%)***				<0.0001
No	2,040	1,841 (99.2)	199 (85.4)	
Yes	49	15 (0.8)	34 (14.6)	
**Elective admission:** ***n (%)***				<0.0001
No	1,755	1,526 (82.2)	229 (98.3)	
Yes	334	330 (17.8)	4 (1.7)	
**Mechanical ventilation in first hour of admission:** ***n (%)***				<0.0001
No	1,363	1289 (69.5)	74 (31.8)	
Yes	726	567 (30.5	159 (68.2	
(SBP)^2^/1000: mean (SD)	2,087	10.67 (4.93)	8.4 (5.01)	<0.0001
FiO_2_ as a decimal: mean (SD)	2,071	0.42 (0.24)	0.63 (0.27)	<0.0001
PaO_2_ (mmHg): mean (SD)	2,058	93.55 (48.85)	88.18 (65.59)	0.132
100*FiO_2_/PaO_2_: mean (SD)	2,087	0.59 (0.62)	1.09 (1.07)	<0.0001
**Bypass cardiac procedure:** ***n (%)***				1
No	2,079	1,847 (99.5)	232 (99.6)	
Yes	10	9 (0.5)	1 (0.4)	
**Non-bypass cardiac procedure:** ***n (%)***				0.8441
No	2,079	1,825 (98.3)	228 (97.9)	
Yes	10	32 (1.7)	5 (2.1)	
**Non-cardiac procedure:** ***n (%)***				<0.0001
No	1,765	1,543 (83.1)	222 (95.3)	
Yes	324	313 (16.9)	11 (4.7)	
**Very high risk diagnosis:** ***n (%)***				<0.0001
No	1,952	1,787 (96.3)	165 (70.8)	
Yes	137	69 (3.7)	68 (29.2)	
**High risk diagnosis:** ***n (%)***				<0.0001
No	2,028	1,812 (97.6)	216 (92.7)	
Yes	61	44 (2.4)	17 (7.3)	
**Low risk diagnosis:** ***n (%)***				<0.0001
No	1,838	1,611 (86.8)	227 (97.4)	
Yes	251	245 (13.2)	6 (2.6)	

*SD, Standard deviation; SBP, Systolic blood pressure; FiO_2_, Fraction of inspired oxygen; PaO_2_, Partial pressure of oxygen in arterial blood*.

### Logistic Regression and Recalibrated PIM3

The tables of coefficients of the logistic regression and recalibrated PIM3 model are presented in Tables 3 and 4, respectively.

**Table 3:** Logistic regression model.

**Variable**	**Coefficients**	**Adj. OR (95% CI)**	***P*-value**
**Logistic regression model**			
Age	0.0033 (−0.0006, 0.0071)	0.9994 (1.0071, 1.002)	0.094
Pupils fixed to light	2.2058 (1.4116, 3)	4.1024 (20.0864, 1.4993)	<0.0001
Elective admission	−1.4421 (−2.4855, −0.3986)	0.0833 (0.6712, 1.7024)	0.0067
Mechanical ventilation in first hour of admission	0.7174 (0.3524, 1.0824)	1.4225 (2.9519, 1.2046)	0.0001
Absolute value of base excess	0.0731 (0.0516, 0.0947)	1.0529 (1.0993, 1.011)	<0.0001
SBP	−0.0081 (−0.0152, −0.001)	0.9849 (0.999, 1.0036)	0.026
SBP*SBP/1000			
FiO_2_ as decimal	1.7802 (1.1211, 2.4392)	3.0682 (11.4644, 1.3994)	<0.0001
PaO_2_ mmHg	−0.0027 (−0.0057, 0.0003)	0.9944 (1.0003, 1.0015)	0.0737
Non–cardiac procedure	−0.7213 (−1.4525, 0.0099)	0.234 (1.0099, 1.4518)	0.053
Very high risk diagnosis	1.5721 (1.11, 2.0342)	3.0343 (7.6458, 1.2657)	<0.0001
High risk diagnosis	1.1497 (0.473, 1.8263)	1.6048 (6.2109, 1.412)	0.0009
Low risk diagnosis	−1.5832 (−2.462, −0.7044)	0.0853 (0.4944, 1.5653)	0.0004

*OR, Odds ratio; CI, Confidence Interval; SBP, Systolic blood pressure; FiO_2_, Fraction of inspired oxygen; PaO_2_, Partial pressure of oxygen in arterial blood*.

**Table 4:** Recalibrated PIM3 model.

**Variable**	**Coefficients**	**Adj. OR (95%CI)**	***P*-value**
**Recalibrated PIM3 model**			
Pupils fixed to light	2.1819 (1.3914, 2.9724)	8.8635 (4.0207, 19.5393)	<0.0001
Elective admission	−1.4844 (−2.518, −0.4508)	0.2266 (0.0806, 0.6371)	0.0049
Mechanical ventilation in first hour of admission	0.8826 (0.5326, 1.2325)	2.4171 (1.7034, 3.4298)	<0.0001
Absolute value of base excess	0.0728 (0.0514, 0.0941)	1.0755 (1.0528, 1.0987)	<0.0001
Systolic Blood Pressure(SBP)	−0.006 (−0.0333, 0.0212)	0.994 (0.9672, 1.0214)	0.6636
SBP*SBP/1000	−0.0169 (−0.165, 0.1313)	0.9833 (0.8479, 1.1403)	0.8233
100*FiO_2_/PaO_2_	0.4527 (0.2592, 0.6462)	1.5725 (1.2959, 1.9082)	<0.0001
Bypass cardiac procedure	−1.3455 (−4.3127, 1.6217)	0.2604 (0.0134, 5.0616)	0.3738
Non-bypass cardiac procedure	−0.1055 (−1.2933, 1.0823)	0.8999 (0.2744, 2.9515)	0.8617
Non-cardiac procedure	−0.7835 (−1.5114, −0.0556)	0.4568 (0.2206, 0.9459)	0.0348
Very high risk diagnosis	1.5912 (1.1336, 2.0488)	4.9096 (3.1067, 7.7589)	<0.0001
High risk diagnosis	1.1528 (0.4834, 1.8222)	3.1672 (1.6216, 6.1857)	0.0007
Low risk diagnosis	−1.6566 (−2.5327, −0.7806)	0.1908 (0.0794, 0.4581)	0.0002

*OR, Odds ratio; CI, Confidence Interval; SBP, Systolic blood pressure; FiO_2_, Fraction of inspired oxygen; PaO_2_, Partial pressure of oxygen in arterial blood*.

### ANN Model

After the selection of hyperparameters, cross validation was undertaken to evaluate model performance on the training data. The cross-validation showed satisfactory discrimination (mean AUROC 0.82, mean AUPRC 0.48) and calibration over 10 folds. The results obtained during the cross-validation phase are shown in Table 5.

**Table 5:** Cross validation performance.

**Variable**	**Mean value**
AUROC	0.82
AUPRC	0.48
Mean squared error	0.07
Calibration slope	1.07
Calibration intercept	0.03

*AUROC, Area under the receiver operating curve; AUPRC, Area under the precision recall curve*.

### Main Outcomes

The plots of performance of the three developed models and PIM3 are shown in Figure 1. The test set AUROC for the ANN models was 0.89 (95% CI = 0.87–0.91), 0.87 (95% CI = 0.85–0.89) for the LR model and 0.86 (95% CI = 0.84–0.88) for the recalibrated PIM3 model. AUPRC was higher for the ANN at 0.60 (95%CI = 0.57–0.64) compared to LR which had an AUPRC of 0.53 (95% CI = 0.50–0.57) and recalibrated PIM3 which had an AUPRC of 0.58(95% CI = 0.55–0.62). The mean squared error for both the ANN and LR models was 0.07. The AUPRC baseline for a random classifier is 0.1. The mean squared error was 0.06 for the recalibrated PIM3 model. The LR and recalibrated PIM3 models were weakly calibrated in terms of the hierarchy proposed by Van Calster et al. (34). The slope and intercept of the flexible calibration curve of the LR model were 1.09 (95% CI 0.66–1.51) and 0.05, respectively. The slope and intercept of the recalibrated PIM3 model was 1.01 (95% CI 0.72–1.31) and 0.01, respectively. The ANN was moderately calibrated. The slope and intercept of the flexible calibration curve of the ANN model were 1.12 (95% CI =1.002–1.23) and 0.01 respectively. The flexible calibration curve of the ANN model, however, is consistently close to the diagonal. The HL *p*-value the ANN, LR and recalibrated PIM3 models was not significant at 0.34, 0.49 and 0.18, respectively. We evaluated the performance of PIM3 over the data set (an external validation). The AUROC for PIM3 was 0.84, the AUPRC was 0.46, the slope of the calibration curve was 0.64 (95%CI = 0.34–0.97) with an intercept of 0.16. PIM3 tends to underestimate risk in the lower probability range, and overestimate risk in the higher probability range. The HL *p*-value from PIM3 across the data set was <0.0001. In lay terms, all three models demonstrate ability to discriminate between survivors and non-survivors with a very modest advantage in favor of the ANN model but the small number of non-survivors compared to survivors makes the AUROC overly optimistic. The AUPRC better evaluates the performance of the models on non-survivors and shows that while the models are not perfect classifiers, they perform significantly better than a random classifier model. The probabilities of death generated by the ANN model appear to be more closely related to the real probability than in the case in the LR and recalibrated PIM3 models.

## Discussion

In this study, we have developed an ANN model for the prediction of mortality prior to discharge from the PICU in two centers in a LMIC and compared it to both a logistic regression model developed on the same data set as well as a recalibrated version of PIM 3. This was achieved using freely usable software without the use of specialized computer infrastructure. The use of a simple feed forward perceptron architecture was able to achieve an effective predictive model without the need for advanced computer hardware, which is a relevant consideration when developing and deploying such models in LMICs. We have demonstrated favorable characteristics of the ANN model compared to LR and PIM3, especially in its performance on the positive class with higher area under the precision recall curve.

Our findings support the need to report model performance comprehensively and transparently (35, 47). The effect of class imbalance in binary classification models was also relevant to the performance of the ANN model. We found the AUROC to be an optimistic measure of model performance in this study due the degree of class imbalance in the data set (29). Despite the similarity in the AUROC between the ANN and the developed LR model in this study, the performance of the ANN assessed by the precision recall curve is superior to the LR and recalibrated PIM3 models. Assessment of calibration by means of the HL statistic alone is not adequate as it does not provide detail on calibration performance. We have found the hierarchy of calibration suggested by Van Calster et al. to be an appropriate method for assessing model calibration which provides more insight into the performance of the model than the HL statistic alone (34). Despite the similar slopes and intercepts for the ANN and LR models, the inspection of the calibration plot suggests that the predictions of the ANN model are more closely related to true probabilities than the LR model.

The model developed in this study is less sophisticated than those described by Kim et al. (24) and Aczon et al. (21, 23). Kim et al. made use of a deep learning model (convolutional neural networks) together with a combination of static and temporal data, extracted from electronic health records, to make real time predictions of mortality (24). Aczon et al. have reported the development of a recurrent neural network model for continuous prediction of PICU mortality. Their recurrent neural network model demonstrates the ability of recurrent neural networks to use data with many variables and time series to make robust predictions. Using electronic health records, Aczon et al. were able to extract 430 distinct variables and use these to develop a highly accurate model for mortality prediction with a higher AUROC than the Pediatric Index of Mortality 2, Pediatric Risk of Mortality III and Pediatric Logistic Organ Dysfunction (23). The ability to process hundreds of variables and interpret them continuously within an automated system in real time would certainly constitute a major advance from existing models and has the potential to provide decision support to clinicians. However, until such a time as the necessary infrastructure and resources are available to investigate such sophisticated models in LMICs, there is a need to investigate the optimal use of available data and technology as demonstrated in this study.

In LMICs there is a need to expand machine learning and other data science research, not only in clinical research but in scholarship in general. Whereas, previous periods of technological advancement were linear in nature, the current period (the Fourth Industrial Revolution) is characterized by an accelerating (exponential) growth in the sophistication of technology. This has raised concerns that those with means to respond to these technologies will draw disproportionate benefit. This could significantly exacerbate existing inequalities (48). Ayentimi and Burgess have suggested that lack of necessary skills and government investment in education in sub-Saharan Africa will likely limit the ability of this region to participate and benefit from the current technological revolution (49). The need to prevent such widening inequality extends to all spheres of scholarship in LMICs, including biomedical research. Where the numbers of neonatal and child deaths remain the highest in sub-Saharan Africa and South Asia (50), medical scholarship in this region must also participate in efforts to prevent a widening of this technological gap. Through the exploration of the use of ANNs and, more broadly, machine learning in this study, we have gained valuable experience and encountered unique challenges in our setting that may guide the development of further research questions.

The lack of accessible health records in our centers have limited the scope of this exploration. This is in keeping with the findings of Katurura and Cilliers, who identified technical, social, and environmental barriers to the successful implementation of electronic health records in South Africa (46). This has limited our study to variables of PIM3. This limits the degree to which ANNs can discover new features, non-linearity and complex relationships that are not evident to logistic regression models. It has also limited us to the investigation of static variables. Furthermore, the small size of the available data set has been a significant barrier in this process. This has both limited the performance of the developed model and necessitated the need to integrate methods to conserve data without allowing the leakage of data from the test set to the training data. We have attempted to overcome this using cross-validation for the hyperparameter tuning and training phase. While our study does not investigate novel variables, it does demonstrate that ANN models can feasibly be developed from data from patients in our setting. The favorable performance of the ANN model serves as a useful proof of concept to underpin future planned prospective research where a wider range of variables as well as methods for enhancing data availability in LMICs can be investigated.

The collection of data during documented clinical care is a key component of the learning healthcare system which can be defined as:

“A learning healthcare system is one that is designed to generate and apply the best evidence for the collaborative healthcare choices of each patient and provider; to drive the process of discovery as a natural outgrowth of patient care; and to ensure innovation, quality, safety, and value in health care” (51).

The South African National Department of Health has published its National Digital Health Strategy for South Africa 2019–2024 (https://www.health.gov.za/wp-content/uploads/2020/11/national-digital-strategy-for-south-africa-2019-2024-b.pdf). Intended to integrate within the planned National Health Insurance scheme, this policy has identified priorities for digital health in South Africa. These include the need to develop a complete electronic health record for all patients, the digitization of health systems and the need to develop digital health knowledge (52). This is in line with the challenges identified by Katurura and Cilliers (53) and will likely enhance the availability of data for machine learning and other data driven research if properly implemented. These priorities must go beyond service delivery and clinical care and be integrated with research, innovation, and discovery such as the investigation of machine learning within this framework, if the full benefit of a learning healthcare system is to be realized in South Africa. The findings of our study demonstrate that machine learning research can be meaningfully conducted in our setting and support a need to integrate machine learning within this policy framework.

We have found Python 3 to be a feasible platform for use by clinician researchers in this setting, who were previously unfamiliar with programming. Python syntax is easily understood and implemented. There are a wide range of integrated algorithms within the available libraries which limit the need for complex programming and calculations (54). The availability of these powerful tools on an open-source platform is a strong argument for the use of Python in our setting where costs are an important factor. Combined with the ability of neural networks to learn complex relationships from data with relatively low-level programming.

From the finding of this study and the discussion above, it is critical that this research be developed further and expanded upon. External validation studies of this model are planned both in other centers in South Africa, but also in other African nations. They also, however, point to priority research that is needed to develop machine learning research and implementation in South Africa and LMICs. Further research is planned to collect, prepare, store and curate large, complex clinical data sets for application in machine learning in these settings. These data include static data with a wider range of features, time series data, medical images and monitoring device data. There is also a need for research into integrating documented clinical care into research activity and promoting the development of systems for EHR in resource limited settings. This research requires the development of networks of pediatric critical care providers across Africa as well as between clinical researchers, computer scientists and engineers, information technology infrastructure experts, biostatisticians and policy makers.

## Conclusion

While this study does not represent a generalized shift in the approach to mortality prediction, it does engage with a widening spectrum of tools and contribute to progress in this field, particularly in LMICs. ANN models are a feasible methodology for such modelling in our setting and can be developed with commercially available computers and free to use software. The ANN, LR and recalibrated PIM3 models showed good predictive performance in terms of the AUROC. The PRC demonstrated that models were significantly better than random classifiers but also significantly below the level of a perfect classifier. The ANN demonstrated an advantage in terms of the precision recall curve and calibration. The true performance of the developed models should be assessed comprehensively in an external validation study. The exploration of the full potential of ANN models was limited by lack of electronic health records and a limited data set. The presence of more sophisticated models such as that of Aczon et al. (21, 23) in the literature suggest that the developed model likely does not fully explore the power of ANNs, particularly for data with many variables and time series data and suggest that further prospective research should be undertaken in our setting. Despite these limitations, the performance of the ANN model in this study is an important proof of concept, demonstrating that ANN and other machine learning models can be developed in LMICs with efficient use of resources. This opens a wide range of research questions and informs the design and execution of clinical machine learning research in South Africa and LMICs. The key next step in the development of this research is the establishment of a large, complex data set from South Africa and LMIC settings to facilitate machine learning research in this context.

## Data Availability Statement

The raw data supporting the conclusions of this article will be made available by the authors, without undue reservation.

## Ethics Statement

The studies involving human participants were reviewed and approved by Health Sciences Research Ethics Committee Affiliated to the University of the Free State. Written informed consent from the participants' legal guardian/next of kin was not required to participate in this study in accordance with the national legislation and the institutional requirements.

## Author Contributions

MP was the primary investigator and was responsible for conceptualization, protocol design, data collection, development of the artificial neural network model, design of figures, and manuscript writing. JS provided inputs into the study protocol, performed the statistical analysis, developed the logistic regression mode, recalibrated PIM3 model, and provided inputs into the manuscript. NL provided inputs into the study protocol, assisted with the development of the artificial neural network model, and provided inputs into the manuscript. LS provided inputs into the study protocol, data collection, development of the logistic regression model, and manuscript. All authors contributed to the article and approved the submitted version.

## Funding

The research was conducted using funds from the National Research Foundation of South Africa. MP is the grantholder under a Thuthuka PhD facility TTK200412512685. An Interdisciplinary Research Grant from the University of the Free State was also provided to assist with this research.

## Conflict of Interest

The authors declare that the research was conducted in the absence of any commercial or financial relationships that could be construed as a potential conflict of interest.

## Publisher's Note

All claims expressed in this article are solely those of the authors and do not necessarily represent those of their affiliated organizations, or those of the publisher, the editors and the reviewers. Any product that may be evaluated in this article, or claim that may be made by its manufacturer, is not guaranteed or endorsed by the publisher.
